# Immune Checkpoint Blockade in Cancer Treatment: A Double-Edged Sword Cross-Targeting the Host as an “Innocent Bystander”

**DOI:** 10.3390/toxins6030914

**Published:** 2014-03-03

**Authors:** Lucia Gelao, Carmen Criscitiello, Angela Esposito, Aron Goldhirsch, Giuseppe Curigliano

**Affiliations:** Division of Early Drug Development for Innovative Therapies, Istituto Europeo di Oncologia, Via Ripamonti 435, Milano 20141, Italy; E-Mails: lucia.gelao@ieo.it (L.G.); carmen.criscitiello@ieo.it (C.C.); angela.esposito@ieo.it (A.E.); aaron.goldhirsch@ieo.it (A.G.)

**Keywords:** checkpoint-blocking antibodies, immune response, toxicity, immune therapies, ipilimumab

## Abstract

Targeted immune checkpoint blockade augments anti-tumor immunity and induces durable responses in patients with melanoma and other solid tumors. It also induces specific “immune-related adverse events” (irAEs). IrAEs mainly include gastrointestinal, dermatological, hepatic and endocrinological toxicities. Off-target effects that arise appear to account for much of the toxicity of the immune checkpoint blockade. These unique “innocent bystander” effects are likely a direct result of breaking immune tolerance upon immune check point blockade and require specific treatment guidelines that include symptomatic therapies or systemic corticosteroids. What do we need going forward to limit immune checkpoint blockade-induced toxicity? Most importantly, we need a better understanding of the roles played by these agents in normal tissues, so that we can begin to predict potentially problematic side effects on the basis of their selectivity profile. Second, we need to focus on the predictive factors of the response and toxicity of the host rather than serially focusing on individual agents. Third, rigorous biomarker-driven clinical trials are needed to further elucidate the mechanisms of both the benefit and toxicity. We will summarize the double-edged sword effect of immunotherapeutics in cancer treatment.

## 1. Introduction

The primary concept of cancer immunotherapy is to enable the immune system to detect neoplastic growth and to either prevent carcinogenesis and/or reject transformed cells with a potential for malignant tumor growth. Immunotherapy in cancer, and, especially, the implementation of active immunotherapy into clinical trials (specifically in the adjuvant setting), has been a largely frustrating experience over the last two decades. Recent advances in clinical and basic research led to a new understanding of the immunology and heterogeneity of cancer. Chemotherapy and targeted treatments can modulate the immune system. Immune response to cancer is a dynamic process that can lead to the rejection of cancer, but can also have regulatory effects that promote tumor growth. The concept of immunoediting in cancer has profoundly changed our current knowledge about the long-term efficacy of chemotherapy and radiotherapy [1]: in fact, treatments formally believed to be highly immunosuppressive can potentially enhance immune response.

## 2. Role of Immunotherapy in Cancer Treatment

Evading immune destruction should be considered an emerging hallmark of cancer. The knowledge of the underlying principles of tumor biology and immunology, enhanced by recent insights into the mechanisms of immune recognition, regulation and tumor escape, has provided new approaches for cancer immunotherapy [2]. Highly immunogenic cancer cells can be eliminated in immunocompetent hosts as a result of the ‘‘immunoediting’’ process. Weakly immunogenic variants can grow and generate solid tumors [1].

A variety of tumor infiltrating cells, including regulatory T-cells (Tregs), myeloid-derived suppressor cells (MDSC) and activated (type 2) macrophages (M2), are involved in the modulation of immune responses in cancer patients [3]. For example, an increased number of Tregs was found in blood and in the tumor microenvironment of patients affected by different tumors: it was demonstrated that Tregs suppress T-cell response and natural killer (NK) cell proliferation and function, thus interfering both with acquired and innate immunity [4]. The prognostic significance of tumor infiltration by Tregs is yet unclear. In some tumor types, including ovarian and breast cancer, an increased number of intratumoral Tregs is associated with bad prognosis [5,6], whereas in other types, such as colorectal cancer and head and neck carcinoma tumor, infiltration by Tregs frequently correlates with improved disease outcome [7,8].

Therefore, cancer tries to evade the immune system by exploiting a series of immune escape mechanisms that were developed to avoid autoimmunity (mechanisms of tolerance). Among these mechanisms are the hijacking of immune cell-intrinsic checkpoints that are induced on T-cell activation. The blockade of one of these checkpoints such, as cytotoxic T-lymphocyte-associated antigen 4 (CTLA-4) [9] or the programmed death 1 (PD-1) receptor, recently provided the first evidence of the activity of an immune-modulation approach in the treatment of solid tumors [10,11]. Several efforts have also been made in recent years to identify other molecules involved in the immune response to develop a wide variety of potential immunotherapeutic targets for the treatment of cancers [12]. Some approaches use antibodies against a specific tumor-associated antigen (TAA) or T-lymphocytes taken from cancer patients and then modified with genes encoding receptors that recognize cancer-specific antigens (passive immunotherapy) [13,14]. Other approaches employ TAAs that are injected into the host with dendritic cells (DCs) or adjuvants to develop a specific anti-tumor immune response (active immunotherapy) [15]. The majority of these approaches have provided encouraging results, inducing a detectable tumor-antigen-specific immunity and, in some case, clinical benefit. However, the potent and specific immune responses generated by some of these immunotherapeutic strategies did not obtain a prolonged objective responses in cancer patients. Several reasons may explain these unsatisfactory results and the difficulty to develop effective immunotherapies in controlling cancer. One reason might be that many antigens identified as therapeutic targets in human cancer are self or “self-altered” antigens, which are aberrantly expressed or overexpressed on transformed cells. In order to develop a specific and long-lasting immune response and increase the success of immunotherapy, it might be useful to disrupt the immune-regulatory mechanisms that contribute to tumor tolerance [16]. Given these observations, several modalities have been developed to target immune suppressive components, such as depletion of Treg [17,18], inhibition of immune suppressive metabolites, including indolamine-2,3-dioxygenase (IDO), arginase and inducible nitric oxide synthetase (iNOS) [19], or targeting immune inhibitory molecules, such as signal transducer and activator of transcription 3 (STAT-3) [20]. The immune checkpoint blockade targeted agents, such as ipilimumab, anti-CTLA-4, anti-PD-1 receptor or anti-programmed death-1 ligand-1 (PD-L1), can be considered really breakthrough drugs in the treatment of solid tumors, and the use of checkpoint blockade antibodies has generated great enthusiasm [21]. Finally, agonistic monoclonal antibodies targeting co-stimulatory molecules, including cluster of differentiation (CD)-40, CD-134 and CD-137, have been developed and evaluated in Phase I clinical trials for solid and hematological malignancies [22,23].

## 3. Mechanisms of Action of Immunomodulators

The activity of a T-cell is regulated by the expression of various molecules and a variety of immuno-modulatory signals, both co-stimulatory and co-inhibitory, that are required to generate an optimal antigen-specific immune response [14].

**Figure 1:**
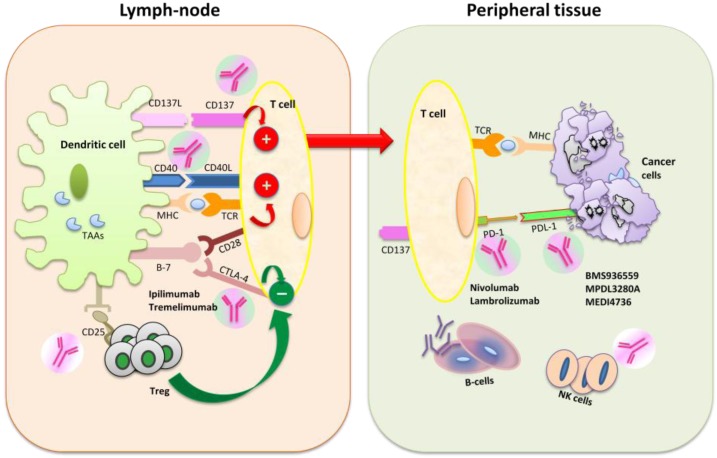
The ‘‘two-signal’’ model of T-cell activation, first requiring the interaction of T-cell receptor (TCR) with a major histocompatibility complex (MHC) molecule expressed by antigen presenting cells (APCs). To complete T-cell activation, the interaction of the CD28 receptor on T-cells with B7 co-stimulatory molecules (B7-1 and B7-2) on APCs is necessary. This phase occurs primarily within the lymph nodes. To prevent inappropriate T-cell activation, negative regulators of T-cell immunity, including CTLA-4 and PD-1, are required. CTLA-4 competes with CD28 for the interaction with B7, and it is upregulated shortly after T-cell activation. Anti-CTLA-4 antibodies, such as ipilimumab and tremelimumab, block CTLA4 and, thereby, enhance antitumor activity. The PD-1 inhibitory receptor plays an important role in modulating T-cell activity in the peripheral tissues during the effector phase. The ligation of PD-1 with PD-L1 causes the negative regulation of T-cells in the tumor microenvironment. Blockade with antibodies of PD-1 or PD-L1 (e.g., nivolumab and MK-3475) results in the activation of T-cells. TAA, tumor-associated antigen; NK, natural killer.

In the ‘‘two-signal’’ model of T-cell activation, antigen-specific T-cell activation needs two signals between T-cells and antigen presenting cells (APCs): the first signal involves the presentation of an antigen to a T-cell receptor (TCR) by a major histocompatibility complex (MHC) molecule on APCs. To complete T-cell activation, a second signal is needed and requires the interaction of the CD28 receptor on T-cells to B7 co-stimulatory molecules (B7-1 and B7-2) on APCs [24]. Besides these co-stimulatory signals, negative regulators of T-cell immunity, including CTLA-4 and PD-1, are needed in order to prevent inappropriate T-cell activation, resulting in autoimmunity. Preclinical data suggest that the blockade of these co-inhibitory molecules or enhancement of co-stimulatory molecules can amplify T-cell responses against tumors [21]. Figure 1 summarizes the mechanisms of activation of the immune system following exposure to tumor antigens.

CTLA-4 is a member of the CD28:B7 immunoglobulin superfamily, and it is normally expressed at low levels on the surface of naive effector T-cells and Tregs [25]. After stimulation of a naive T-cells through the TCR, CTLA-4 is upregulated and competes with CD28 for B7 and, finally, leads to suppression of T-cell activity [26]. The induction of tolerance in antigen specific T-cells can be promoted also by other mechanisms, such as direct inhibition of TCR signals, reduction of IL-2 production and downregulation of IL-2 receptor expression [27,28]. Moreover, some studies suggest that the antitumor effect of CTLA-4 blockade might depend on the depletion of Treg [29], as demonstrated in a model of mouse melanoma, in which both the enhancement of T effector cell function and inhibition of Treg activity through the blockade of CTLA-4 led to a strong antitumor response [25].

In addition to CTLA-4, PD-1 is a key immune checkpoint protein and represents a promising immunotherapeutic target. It is a co-inhibitory molecule expressed on chronically stimulated T-cells, as well as Tregs, activated B-cells and natural killer (NK) cells [30]. PD-1 appears to play a crucial function in modulating T-cell activity in peripheral tissues through interaction with its ligands, PD-L1 (B7-H1) and PD-L2 (B7-DC) [10]. PD-L1 and, to a lesser extent, PD-L2 are expressed on many hematologic and non-hematologic human tumors [31]. The evidence of the involvement of the PD-1/PD-L1 pathway in immunosuppression mechanisms arises from experimental models showing that mice with a genetic deficiency of PD-1 present enhanced immunity with phenotypes characterized by autoimmune cardiomyopathy and a lupus-like syndrome [32,33]. In human cancer, the interaction between PD-1 and its ligand, PD-L1, leads to the suppression of T-cell activity, resulting in immune evasion by cancer cells [25]. Since monoclonal antibodies can block this interaction, they have been evaluated as a strategy to augment the immune response.

Beside the CTLA-4 and PD-1/PD-L1 molecules, other immune-modulatory targets have been identified, such as killer immunoglobulin-like receptors (KIRs) expressed by NK cells [34] and the TNF super family co-stimulatory molecules, including CD-40, CD-134 and CD-137 [35,36]. CD40 is expressed by immune cells and by various type of cancer cells. Some studies report that CD40 expression on certain tumor cell types has been implicated in pro-apoptotic and anti-proliferative activity, suggesting a potential use of this target in anticancer treatment. Similarly CD137, expressed by activated T-cells, dendritic and NK cells, seems to enhance T-cell proliferation and IL-2 secretion and might be used to increase immune activity to eliminate tumors.

## 4. Clinical Trials with Immune Checkpoint Blockade Targeted Agents

Based upon the results of preclinical studies that support the evidence of the involvement of these molecules in immune control, various antibodies blocking CTLA-4, PD-1 or PD-L1 or other immune targets are actually used in clinical practice.

Ipilimumab (Yervoy^®^) is a monoclonal antibody (mAb) designed to block CTLA-4, thereby preventing the development of tolerance and augmenting anti-tumor responses [37]. This drug was evaluated in several Phase I/II/III clinical trials and in different tumor types, including prostate cancer, non-small cells lung cancer (NSCLC), renal carcinoma and pancreatic cancer [38,39,40,41]. The efficacy and safety of ipilimumab was most frequently studied in melanoma. The study that led to the approval of ipilimumab by the U.S. Food and Drug Administration (FDA) was a three-arm randomized trial comparing the combination of ipilimumab with gp100 peptide vaccine *versus* gp100 vaccine alone *versus* ipilimumab alone in 676 patients affected by metastatic melanoma who had failed prior therapy [42]. The median overall survival (OS) was increased from 6.4 months to 10.0 months with the addition of ipilimumab to gp100 vaccine (*p* < 0.0001), and also, long-term survival rates improved. Severe or potentially life threatening (Grade 3 or 4) adverse events occurred in 10%–15% of patients treated with ipilimumab and in 3% of those treated with gp100 alone. Fourteen deaths related to the study drugs (2.1%) were recorded. In a subsequent Phase III trial, 502 patients with metastatic melanoma that was previously untreated were randomly assigned to dacarbazine with ipilimumab or dacarbazine with placebo. OS was significantly increased in patients assigned to the ipilimumab arm compared to the placebo arm (median 11.2 *versus* 9.1 months) [9]. Grade 3 and 4 adverse events occurred in 56.3% of patients in the ipilimumab plus dacarbazine group compared with 27.5% treated with dacarbazine and placebo. No drug-related deaths were reported in the ipilimumab group. The European Organization for Research and Treatment of Cancer (EORTC) and the Eastern Cooperative Oncology Group (ECOG) have designed two clinical trials National Clinical trial(NCT)00636168 and NCT01274338, respectively) to evaluate the efficacy of this drug in the adjuvant setting of melanoma. Moreover, combinations of ipilimumab with other therapeutic approaches, such as chemotherapy, immunotherapy, including dendritic cell vaccine, or radiotherapy are currently under investigation in several clinical trials [43].

The clinical activity of another CTLA-4-blocking antibody, tremelimumab, was also investigated. Based on the encouraging response obtained in Phase I/II trials [44,45], a Phase III trial was conducted in which previously untreated patients with melanoma were randomly assigned to either tremelimumab or chemotherapy. The results of this study demonstrated durable responses in patients treated with tremelimumab despite the endpoint of improved OS not being reached [46]. Tremelimumab has also been studied in Phase II trials of patients with metastatic colorectal, gastric, esophageal cancers and NSCLC, alone or in combination with other anticancer therapies [47,48,49].

Given the success of targeting this first immune inhibitory checkpoint, Phase I/II studies of mAb against PD-1 and PD-L1 have been performed. Nivolumab, an anti-PD-1 mAb, was evaluated in a Phase I/II study in 296 patients with pretreated NSCLC, prostate cancer, renal cell carcinoma, colorectal cancer and melanoma [10]. Clinical responses were recorded in around 30% of the melanoma patients, but also, among patients with renal cell carcinoma and NSCLC, such response were seen. At the 2013 American Society of Clinical Oncology (ASCO) annual meeting, the long-term follow-up data of patients treated with nivolumab were presented, confirming an excellent durability of nivolumab-induced responses and showing 61% one-year and 44% two-year survival rates in melanoma patients [50]. The adverse effects were less frequent than those observed in patients treated with ipilimumab. The combination of anti-PD1 and anti-CTLA4 was evaluated also in 53 patients, resulting in an improved response rate, without additional toxicity [51]. Lambrolizumab is another anti-PD-1 mAb, which was tested in 135 patients with advanced melanoma. The response rate in patients treated with this agent was 38%, and the responses were durable in the majority of patients [52].

Clinical activity has been observed also with different anti-PD-L1 drugs [11]. Unlike PD-1 antibodies, PD-L1 antibodies spare potential interactions between PD-L2 and PD-1, but additionally block interactions between PD-L1 and CD80, even if the therapeutic significance of these interactions is still unclear [53]. In a Phase 1 trial, anti-PD-L1 therapy produced durable tumor regression (objective response rate: six to 17%) in patients with metastatic NSCLC, melanoma, renal-cell cancer and ovarian cancer; Grade 3 or 4 adverse events occurred in only 9% of patients [11]. Many anti-PD-L1 agents are currently being investigated, such as BMS-936559 and MPDL3280A, and preliminary data indicate that these mAb are safe in multiple tumor types [54,55].

## 5. Clinical Trials with Immune-Stimulatory and Immune-Suppressor Molecules

Finally, several strategies were designed to target co-stimulatory molecules and immune-suppressor metabolites. Small molecule inhibitors blocking IDO have shown efficacy in preclinical models [56], whereas Phase I/II clinical trial are testing strategies to deplete Tregs through the blockade of their CD25 surface receptor. Denileukin diftitox, an IL-2-diptheria toxin fusion protein, was developed as a therapeutic strategy for cutaneous T-cell lymphoma, and clinical responses have been seen also in melanoma [57]. mAbs against CD25, such as daclizumab, were explored in the clinical setting, and early results indicated durable reduction in Treg numbers after a single dose of daclizumab [58]. In a Phase I trial, BMS-663513, a fully human anti-CD137 agonist mAb, was studied with encouraging results in patients affected by solid tumors, including melanoma ovarian, prostate cancer and NSCLC [35]. Instead, a Phase II trial conducted in patients with metastatic melanoma was stopped, due to an unexpected high incidence of Grade 4 hepatitis [59]. Other clinical trials were performed to assess the efficacy of mAb that targets CD40 in hematologic and solid tumor [22,36]. Recently, the efficacy of mAb against OX40 was investigated, which is a costimulatory receptor expressed primarily on activated CD4+ and CD8+ T-cells. A Phase I clinical trial was performed in patients with advanced cancer, and therapy with anti-OX40 mAb showed an acceptable toxicity profile and the regression of at least one metastatic lesion in 12 of 30 patients, although there were no responses according to the Response Evaluation Criteria In Solid Tumors (RECIST) criteria [60]. Table 1 summarizes the major clinical trials with immunomodulators in solid and hematological malignancies.

**Table 1:** Major clinical trials with immunomodulators in solid and hematological malignancies.

Study drug	National Clinical Trial (NCT) Number	Disease	Therapy	Phase	Primary Endpoint
***Anti-CTLA-4 antibodies***					
Ipilimumab					
	NCT01489059	Melanoma	IL-21 + Ipilimumab	I	Safety
	NCT01676649	Melanoma	Ipilimumab + Carboplatin + Paclitaxel	II	Safety
	NCT01498978	Prostate Cancer	Ipilimumab + Androgen Suppression Therapy	II	Efficacy
	NCT01896869	Pancreatic cancer	FOLFIRINOX Followed by Ipilimumab	II	Efficacy
	NCT01363206	Melanoma	Granulocyte Macrophage-Colony Stimulating Factor + Ipilimumab	II	Safety/Efficacy
	NCT01988077	Melanoma	Adoptive T-Cell Transfer + Ipilimumab	II	Safety/Efficacy
	NCT01856023	Melanoma	IL-2 + Ipilimumab	III	Safety/Efficacy
	NCT01822509	Hematologic Malignancies	Ipilimumab After Allogeneic Hematopoietic Cell Transplantation	I	Safety
	NCT01611558	Ovarian Cancer	Ipilimumab	II	Safety
	NCT01450761	Small Cell Lung Cancer	Etoposide + Platinum +/− Ipilimumab	III	Efficacy
	NCT01604889	Melanoma	Ipilimumab +/− INCB024360	I/II	Safety/Efficacy
	NCT01024231	Melanoma	BMS-936558 + Ipilimumab	I	Safety/Efficacy
	NCT01832870	Prostate Cancer	Sipuleucel-T + Ipilimumab	I	Safety/Efficacy
	NCT01524991	Urothelial Carcinoma	Gemcitabine, Cisplatin + Ipilimumab	II	Safety/Efficacy
	NCT01285609	Squamous Non-Small Cell Lung Cancer	Paclitaxel + Carboplatin +/− Ipilimumab	III	Efficacy
	NCT01565837	Melanoma	Ipilimumab + Stereotactic Ablative Radiation Therapy	II	Safety/Efficacy
	NCT01689974	Melanoma	Ipilimumab *vs*. Ipilimumab + Radiotherapy	II	response rates
	NCT01750983	Advanced Cancers	Ipilimumab + Lenalidomide	I	Safety/Efficacy
	NCT01489059	Melanoma	IL-21/Ipilimumab	I	Safety
	NCT01740297	Melanoma	Ipilimumab +/− Talimogene Laherparepvec	I/II	Safety/Efficacy
	NCT01274338	Melanoma	Ipilimumab or High-Dose Interferon Alfa-2b	III	Efficacy
	NCT01738139	Advanced Cancers	Ipilimumab +/− Mesylate	I	Safety/Efficacy
	NCT01827111	Melanoma	Abraxane + Ipilimumab	II	Safety/Efficacy
	NCT01767454	Melanoma	Ipilimumab + Dabrafenib +/− Trametinib	I	Safety
	NCT01860430	Cancer of Head and Neck	Cetuximab + Radiotherapy + Ipilimumab	I	Safety/Efficacy
	NCT01673854	Melanoma	Vemurafenib Followed by Ipilimumab	II	Safety
	NCT01608594	Melanoma (Neoadjuvant)	Ipilimumab + IFN-α2b	II	Safety/Efficacy
	NCT01590082	Melanoma	Doxycycline, Temozolomide + Ipilimumab	I/II	Safety/Efficacy
	NCT01711515	Cervical Cancer	Chemoradiation Therapy + Ipilimumab	I	Safety
	NCT01810016	Melanoma	NY-ESO-1 Vaccine + Ipilimumab	I	Safety/Efficacy
	NCT01896999	Hodgkin Lymphoma	Ipilimumab and Brentuximab Vedotin	I	Safety
	NCT01473940	Pancreatic Cancer	Ipilimumab and Gemcitabine	I	Safety
	NCT01729806	B-Cell Lymphoma	Ipilimumab + Rituximab	I	Safety
	NCT01331525	Small Cell Lung Cancer	Ipilimumab + Carboplatin + Etoposide	II	Efficacy
	NCT00836407	Pancreatic Cancer	Ipilimumab +/− Vaccine Therapy	I	Safety
	NCT01643278	Gastrointestinal Stromal Tumors or Other Sarcomas	Dasatinib and Ipilimumab	I	Safety
	NCT00636168	Melanoma	Melanoma *vs*. placebo	III	Efficacy
Tremelimumab					
	NCT01843374	Mesothelioma	Tremelimumab *vs*. Placebo	II	Safety/Efficacy
	NCT01853618	Liver Cancer	Tremelimumab + Chemoembolization	I	Safety
	NCT01975831	Solid Tumors	MEDI4736 + Tremelimumab	I	Safety
	NCT01103635	Melanoma	Tremelimumab + CP-870,893	I	Safety
***Anti-PD-1 antibodies***					
Nivolumab					
	NCT01783938	Melanoma	Ipilimumab followed by Nivolumab	II	Safety
	NCT01454102	Non-small Cell Lung Cancer	Nivolumab + Chemotherapy or As Maintenance Therapy	I	Safety
	NCT01928394	Solid Tumors	Nivolumab or Nivolumab + Ipilimumab	I/II	Efficacy
	NCT01642004	Squamous Cell Non-small Cell Lung Cancer	Nivolumab *vs* Docetaxel	III	Efficacy
	NCT01844505 NCT01927419	Melanoma	Nivolumab or Nivolumab + Ipilimumab or Ipilimumab	III/II	Efficacy
	NCT01668784	Renal Cell Carcinoma	Nivolumab *vs*. Everolimus	III	Efficacy
	NCT01968109	Solid Tumors	Anti-LAG-3 +/− Anti-PD-1	I	Safety
	NCT01592370	Hematologic Malignancy	Nivolumab	I	Safety
	NCT01721772	Melanoma	Nivolumab *vs*. Dacarbazine	III	Efficacy
***Anti-CTLA-4 antibodies***					
Ipilimumab					
	NCT01629758	Solid Tumors	IL-21+ Nivolumab	I	Safety
MK-3475					
	NCT01295827	Solid Tumor	MK-3475	I	Safety/Efficacy
	NCT01840579	Solid Tumor	MK-3475 + chemotherapy	I	Safety
	NCT01905657	Non-Small Cell Lung Cancer	MK-3475 *vs*. Docetaxel	II/III	Safety/Efficacy
	NCT01866319	Melanoma	MK-3475 *vs*. Ipilimumab	III	Safety/Efficacy
	NCT01848834	Solid Tumor	MK-3475	I	Safety/Efficacy
***Anti-PD-L1 antibodies***					
BMS-936559					
	NCT00729664	Cancer	BMS-936559	I	Safety
MPDL3280A					
	NCT01633970	Solid Tumors	MPDL3280A + Bevacizumab +/− Chemotherapy	I	Safety
	NCT01656642	Melanoma	MPDL3280A + Vemurafenib	I	Safety
	NCT01846416	Non-small Cell Lung Cancer	MPDL3280A	II/III	Safety/Efficacy
	NCT01903993	Non-small Cell Lung Cancer	MPDL3280A *vs*. Docetaxel	II	Safety/Efficacy
***Other immunomodulators***					
BMS-986015 (Anti-KIR)					
	NCT01750580	Cancer	BMS-986015 + Ipilimumab	I	Safety
Daclizumab (anti CD25)					
	NCT01468311	Hodgkin’s Lymphoma	Daclizumab	I/II	Safety/Efficacy
	NCT01307618	Melanoma	Vaccine +/− IL-12 Followed by Daclizumab	II	Safety/Efficacy
BMS-663513 (CD137 agonist)					
	NCT01471210	Non-Hodgkin’s Lymphoma/Solid Tumors	BMS-663513	I	Safety
	NCT01775631	Non-Hodgkin’s Lymphoma	BMS-663513 + Rituximab	I	Safety

## 6. Immune-Related Toxicity of Immune Checkpoint Inhibitors

Toxicity is a major issue for the new cancer immunotherapy. Ipilimumab and other immunomodulatory drugs have been associated with several immune-related adverse events (irAEs); most of them related to the infiltration of highly-activated CD4 and CD8 T-cells and the increased production of inflammatory cytokines in normal tissues [61]. In fact, skin and gut biopsies performed in the sites of irAEs showed that the involved organs were infiltrated with both CD4+ and CD8+ T-cells. Elevated levels of inflammatory cytokines released by activated T-cells were reported in the sera of these patients [59]. This observation, beside the evidence of the rapid resolution of some irAEs after the use of the anti-tumor necrosis factor (anti-TNF) antibody, infliximab, suggested that cytokines may be associated with the development of toxicities related to the use of these agents.

Animal models and clinical trials support a role for CTLA-4 blockade in breaking the tolerance to both human cancer antigens and self-antigens. CTLA-4 blockade in murine cancer models increased the regression of immunogenic tumors [62], but caused depigmentation, thus implying a role for CTLA-4 not only in tumor antigenicity, but also in the suppression of autoimmunity [63]. Histological evaluation of depigmented lesions revealed the infiltration of polymorphonuclear cells and deposition of antibody. Furthermore, CTLA-4-deficient mice died early, about one month after birth, due to lympho-proliferative disease and autoimmunity [64,65]. Other studies have demonstrated that anti-CTLA-4 in animal models led to T-cell-associated autoimmune toxicities, including diabetes, demyelinating lesions, encephalomyelitis and colitis [66,67,68]. The combination of CTLA-4 blockade and an irradiated tumor cell vaccine in a prostate cancer mouse model elicited a potent antitumor response, but prostatitis accompanied by the destruction of epithelium were also reported, indicating that the immune response was, at least in part, directed against normal prostate antigens [69]. Thus, these autoimmune effects suggest that the immune targets for these responses can be represented by normally expressed differentiation antigens and that CTLA-4 blockade is able to break peripheral immune tolerance.

Phan *et al.* reported that the use of anti-CTLA-4 antibody to metastatic melanoma patients resulted in objective cancer regression in three of 14 patients and autoimmune manifestations in six of 14 patients (43%) [70]. In a subsequent study, same authors hypothesized that in patients with metastatic melanoma, CTLA-4 blockade might enhance the antitumor effect of IL-2, since IL-2 stimulates T-cell growth, but has also been implicated in the expansion of Tregs that express cell-surface CTLA-4. Twenty five percent of responders experienced Grade 3/4 autoimmunity attributable to anti-CTLA-4 therapy. In comparison to previous experience, in this study, the incidence of autoimmunity was decreased, maybe due to the supportive effect of IL-2 on Treg cell activation and proliferation [71].

The most common irAEs involve gastrointestinal tract, skin, liver and endocrine system [72]. These effects are reported in up to 60% of patients treated with ipilimumab, with severe toxicities (Grade 3 or 4) in about 10%–15% of patients [42]. They can appear at various times after anti-CTLA-4 treatment. The average timelines for irAEs are 2–3 weeks for dermatologic events, 6–7 weeks for gastrointestinal and hepatic events and nine weeks for endocrine events [73]. The presentation of irAEs can vary from insidious to sudden and can be confused with other known autoimmune conditions. Usually, irAEs were reversible, but in rare cases, they may be severe and life threatening. The most common dermatologic toxicities include maculopapular, erythematous rash or pruritus. Vitiligo can also be seen and is considered a positive prognostic factor in patients with melanoma, as it signals an immune attack on melanocytes. Frequently, irAEs involved gastrointestinal tract. Grade 3/4 diarrhea/colitis was the most frequently observed serious adverse event in clinical trials. Among 198 patients with metastatic melanoma and renal cancer treated with anti-CTLA-4, 21% experienced Grade 3/4 colitis [74], and the mortality in patients who developed autoimmune colitis due to bowel perforation was about 5% [75]. Enterocolitis has been linked to inflammatory bowel disease or graft-*versus*-host disease (GVHD) [74]. Indeed, it was suggested that common antigens expressed by tumor and bowel induces the T-cell infiltration. Another hypothesis is that enterocolitis was generated by cytokine production or dendritic cell activation by CD4 cells of autoimmune origin.

The elevation of serum liver transaminases and/or bilirubin and inflammatory hepatitis ranges from 2% to 9% across different studies [76]. Ipilimumab-induced hepatitis is rare, but can be life threatening. Finally, endocrinopathies, including hypophysitis, hypopituitarism, adrenal insufficiency, hypothyroidism or hypogonadism, were described in patients treated with ipilimumab and can require an accurate differential diagnosis with other causes [77].

Compared to anti-CTLA-4, agents targeting the PD-1/PD-L1 pathway seem to be better tolerated, with a more favorable toxicity profile, emphasizing the distinct biologic features of the two pathways. One reason that could explain the reduced toxicity could be that the PD1/PD-L1 checkpoint interaction takes place peripherally, *i.e.*, at the tumor site, whereas the CTLA4/B7 interaction occurs mostly centrally, *i.e.*, in the lymphoid organs [78]. Most of the toxicity associated with anti-PD-1/PD-L1 was immune related, as well as with anti-CTLA-4 therapy [10,11]. The most frequent adverse events recorded, regardless of causality, were fatigue, decreased appetite, diarrhea, nausea, dyspnea, constipation, vomiting, rash, pyrexia and headache [10]. The Grade 3/4 adverse event rate was 14% in patients receiving nivolumab. Interestingly, one unique and potentially life-threatening toxicity for these agents is pneumonitis, which occurred in 3% of patients, but only 1%–3% developed a Grade 3 or 4 pneumonitis [10,51,52]. No clear relationship was reported between the incidence of this side effect and tumor type, dose level or the number of doses received. In the majority of cases, it was reversible with treatment discontinuation and/or glucocorticoid administration, but three patients died despite the use of infliximab and mycophenolate [10]. Mild infusion reactions were observed in patients receiving anti-PD-L1 treatment, whereas severe adverse effects were infrequently noted [11]. Indeed, irAEs were observed in 39% of patients and included rash, hypothyroidism, hepatitis and, less frequently, sarcoidosis, diabetes mellitus and myasthenia gravis. These adverse events were predominantly of Grade 1 or 2 and were managed with treatment interruption or discontinuation. The Grade 3/4 adverse event rate was 9% in patients receiving BMS-936559 [49] and was managed with glucocorticoids. Table 2 summarizes the main serious adverse effects of checkpoint inhibitors.

**Table 2:** Grade 3–4 serious adverse events of immune checkpoints inhibitors.

Serious Adverse Events (Grade 3 and 4)	Ipilimumab [9*,^42^,^72^,^74^,^76^]	Tremelimumab [44,45,46]	Anti-PD1 (Nivolumab, Lambrolizumab) [3,51 **,^52^]	Anti-PD-L1 (BMS-936559) [4]
*Dermatologic*				
Rash and/or pruritus	3.2%–4%	2.5%–18%	1%–4%	<1%
*Gastrointestinal*				
Diarrhea	4%–5.3%	5%–21%	1%–3%	<1%
Nausea or vomiting	<5%	8%–13%	0	<1%
Colitis	2%–21%	2.1%–18%	2%	
*Endocrine*				
Hypophysitis	0.8	2%	1%	0
Hypothyroidism	0	1%	1%	0
Hypopituitarism	0.8	1%	Non reported	0
Adrenal insufficiency	1.5	1%	0	<1%
*Hepatic*				
Increase in alanine aminotransferase	1.5%–22%	Not reported	1%–7%	0
Increase in aspartate aminotransferase	0.8%–18%	Not reported	1%–6%	<1%
Hepatitis	<3%	1%	Not reported	<5%
*Fatigue*	6%–10%	2%–13%	2%	3%
*Pneumonitis*	Not reported	1%	1%–3%	Not reported

Notes: * In this study, ipilimumab is in combination with dacarbazine; ** in this study, nivolumab is in combination with ipilimumab.

## 7. Toxicity of Other Immunomodulator Agents

A Phase II clinical trial to assess the efficacy of anti-CD137 in melanoma patients was stopped due to severe side effects. The most common toxicities observed with this treatment were fatigue, transaminitis, neutropenia, rash and diarrhea [35]. Liver injury was the most frequent serious adverse event and, based on evidence from animal models, might be associated with increased liver CD8+ T-cell infiltration. Niu *et al.* demonstrated that CD137 agonist can induce immunologic alteration, resulting ultimately in toxicity in various organs, such as liver, lungs, spleen and bone marrow [79]. Indeed, CD137 receptor cross-linking, CD8+ T-cell and the production of TNF-α, IFN-γ and Type I IFN were crucial in inducing these side events. It is not known if T-cells are the direct targets of anti-CD137 mAb or if they are indirectly influenced by either cytokines or chemokines produced by other lineages of CD137-expressing cells [80].

Nonetheless, the evidence that in absence of liver-infiltrating CD8+ T-cells or of TNF-α, anti-CD137 injected mice do not develop hepatitis supports the hypothesis that cytokine-induced inflammation was one of the causes of liver pathology. Thus, CD137-targeted immunostimulation, despite encouraging results with regard to immune responses, needs further evaluation to find a safer dosing with tolerable liver toxicity.

The Phase I study that evaluated the safety and antitumor activity of dacetuzumab (anti-CD40 agent) in 44 patients with advanced multiple myeloma demonstrated that the treatment was generally well tolerated, and the most frequent adverse events potentially related to dacetuzumab were represented by cytokine release syndrome symptoms, non-infectious ocular inflammation and elevated hepatic enzymes [81]. These events were observed in 11%–16% of patients, and only 8% of them reported serious toxicity. A similar toxicity profile was observed in Phase I trials in patients with chronic lymphocytic leukemia and non-Hodgkin’s lymphoma [82,83]. The results of ongoing clinical trials are necessary to define the exact toxicity profile of this drug.

Daclizumab, an anti-CD25 antibody, indicated in multiple sclerosis and to prevent rejection in organ transplantation, was studied in breast cancer patients with regard to human Treg survival and function. In this study, daclizumab, combined with an experimental cancer vaccine, led to a marked and prolonged decrease in Tregs in patients. Autoimmune reaction, although expected, was not observed in this study [84]. However, further studies are needed to evaluate the antitumor activity of this approach and related toxicities.

## 8. Toxicity: Management and Correlation with Outcome

The management of irAEs are based upon the severity of the observed toxicity and typically involve the early detection of toxicity, interruption of therapy, close clinical monitoring and early symptomatic relief. Not all irAEs will require permanent cessation of therapy. The primary treatment for most low-grade irAEs is supportive care, including oral hydration and loperamide for diarrhea, antipruritic medications or topical steroids for dermatologic lesions. Administration of systemic corticosteroids is required in the case of Grade 3–4 toxicities and is usually associated with the effective and rapid reversal of symptoms [30]. Despite the theoretical concern that corticosteroids or immunomodulators may blunt the antitumor effect of therapy, corticosteroids do not appear to affect the efficacy of anti-CTLA-4 therapy, and antitumor activity continues to be recorded even in patients using steroids [85]. If symptoms persist despite high-dose steroids or are refractory to steroid tapering, other immunosuppressive treatments may be necessary, such as infliximab for severe enterocolitis [85]. Algorithms have been developed to aid in the management of these side effects and are available at the FDA Risk Elimination and Management System (REMS) website [86]. Utilizing these management strategies, life-threatening complications have been minimized.

Interestingly, data derived from several early clinical trials suggested a possible relationship between irAEs and the clinical benefit of therapy, since patients who developed irAEs have shown a higher ratio of therapeutic response [85,87]. In a trial in which 56 Stage IV melanoma patients were treated with ipilimumab and a peptide vaccine, 36% of patients who had a severe irAE achieved a clinical response, whereas only 5% of patients without an irAE showed a response [88]. Similar results were observed in a double-blind, placebo-controlled Phase II study in which the response rate was higher in patients who experienced Grade 3/4 irAEs than in patients with Grade 0–2 irAEs [89]. Thus, the severity of irAE could be a surrogate marker of drug efficacy, even if the meaning of the relationship between the severity of irAEs and clinical response needs further confirmation to be considered definitive.

## 9. Conclusions

Breaking immune tolerance upon immune check point blockade may induce durable cancer proliferation control and, on the other hand, off target side effects affecting normal tissues as “innocent by-stander”. A better knowledge of the irAEs, as well as their management and prevention, is of enormous significance for a proper diagnosis and, accordingly, therapy. If a link between these AEs and specific immunotherapeutics is recognized, the withdrawal of therapy might be envisaged.

A better understanding of the mechanisms of action of these drugs and of their interaction with the immune system and normal tissues might be useful to improve the toxicity profile with no impact on clinical response.

Several questions are raised by all the data presented in this review article; the answer to these questions should be considered a possible area of research in the following years. Which patients should be treated with an immunotherapy approach? It is possible to predict the side effects developed during the course of immune-therapy? Is there any genetic signature predicting response to immunotherapy? What are the risks associated with such a treatment, *i.e.*, the possibility of developing an autoimmune response? What is the durability of immune protection? Can we combine immune checkpoint blockade therapy with immunosuppressors to reduce side effects? Which are the optimal biological doses, schedules, methods of administration, timing and potential following boosts to maintain a durable immune response? Another important long-term concern for trials ongoing in the adjuvant setting is the potential induction of autoimmunity, which depends on the kind of tumor antigen that is targeted and the response that is elicited. Long-term follow up of these patients is a major issue. We need a better understanding of the relation between innate and adaptive immune responses and of the immune escape mechanisms employed by tumor cells, the discovery of the mechanisms underlying immunological tolerance and acknowledgment of the importance of both cell-mediated and humoral adaptive immunity for the control of tumor growth. What do we need going forward to limit immune checkpoint blockade-induced toxicity? Most importantly, we need a better understanding of the roles played by these agents in normal tissues, so that we can begin to predict potentially problematic side effects on the basis of their selectivity profile. Second, we need to focus on the predictive factors of response and toxicity of the host rather than serially focusing on individual agents. Third, rigorous biomarker-driven clinical trials are needed to further elucidate the mechanisms of both the benefit and toxicity.
